# Diagnostic performance of a computer-assisted diagnosis system for bone scintigraphy of newly developed skeletal metastasis in prostate cancer patients: search for low-sensitivity subgroups

**DOI:** 10.1007/s12149-017-1175-2

**Published:** 2017-04-29

**Authors:** Mitsuru Koizumi, Kazuki Motegi, Masamichi Koyama, Takashi Terauchi, Takeshi Yuasa, Junji Yonese

**Affiliations:** 1 Department of Nuclear Medicine, Cancer Institute Hospital, 3-8-31 Ariake, Koto-ku, Tokyo, 135-8550 Japan; 2 Department of Urology, Cancer Institute Hospital, Tokyo, Japan

**Keywords:** Computer-assisted diagnosis, Bone scintigraphy, Bone metastasis, Prostate cancer

## Abstract

**Purpose:**

The computer-assisted diagnostic system for bone scintigraphy (BS) BONENAVI is used to evaluate skeletal metastasis. We investigated its diagnostic performance in prostate cancer patients with and without skeletal metastasis and searched for the problems.

**Methods:**

An artificial neural network (ANN) value was calculated in 226 prostate cancer patients (124 with skeletal metastasis and 101 without) using BS. Receiver operating characteristic curve analysis was performed and the sensitivity and specificity determined (cutoff ANN = 0.5). Patient’s situation at the time of diagnosis of skeletal metastasis, computed tomography (CT) type, extent of disease (EOD), and BS uptake grade were analyzed. False-negative and false-positive results were recorded.

**Results:**

BONENAVI showed 82% (102/124) of sensitivity and 83% (84/101) specificity for metastasis detection. There were no significant differences among CT types, although low EOD and faint BS uptake were associated with low ANN values and low sensitivity. Patients showed lower sensitivity during the follow-up period than staging work-up. False-negative lesions were often located in the pelvis or adjacent to it. They comprised not only solitary, faint BS lesions but also overlaying to urinary excretion.

**Conclusions:**

BONENAVI with BS has good sensitivity and specificity for detecting prostate cancer’s osseous metastasis. Low EOD and faint BS uptake are associated with low sensitivity but not the CT type. Prostate cancer patients likely to have false-negative results during the follow-up period had a solitary lesion in the pelvis with faint BS uptake or lesions overlaying to urinary excretion.

## Introduction

Prostate cancer is the most common solid cancer in men in the US [1] and the third most common in Japan, and its incidence is increasing [2]. Bone is the most common site of distant metastases of prostate cancer [3]. These skeletal metastases are usually osteoblastic in contrast to those from other cancers, which are mostly osteolytic [4]. Therefore, bone scintigraphy (BS) is used to diagnose skeletal metastasis from prostate cancer. To date, BS has been interpreted visually. Therefore, there was a need for an appropriate quantitative approach for BS, and computer-assisted diagnostic (CAD) software for BS was developed and evaluated [4–8]. A Japanese version of this software is BONENAVI. We formally published the development and clinical evaluation of a revised version, called BONENAVI II [9]. As above mentioned, skeletal metastases of prostate cancer are mostly osteoblastic, other types of skeletal metastasis exist. Our clinical questions concerned the performance of BONENAVI with the various types of skeletal metastasis and what factors, if any, caused the failures (false-negative and false-positive results) to diagnose skeletal metastases from prostate cancer.

We conducted this study to clarify the diagnostic performance of BONENAVI for detecting newly developed skeletal metastases from prostate cancer as an adjunct to the clinical information, BS results, and computed tomography (CT) appearance and then analyzed the factors as to how they related to the diagnostic failures. Identifying the factors and the reasons they produce a low artificial neural network (ANN) value and low sensitivity may help improve the performance of CAD software.

## Materials and methods

### Patients

Between February 2013 and January 2017, consecutive patients with histologically proven prostate cancer and who showed typical or suspected skeletal metastases on BS were enrolled. These patients were reviewed regarding the presence of skeletal metastasis, and the both BS and CT scan of the osseous metastatic sites were performed at the time of the first diagnosis of skeletal metastasis, which was established by agreement of at least two different modalities, follow-up studies, and/or biopsy.

Eligible patients had a diagnosis of skeletal metastasis and the interval between BS and CT scan was within a month. These patients were then divided into the following diagnostic groups depending on the time of staging and the follow-up period after initial therapy. The follow-up period was further divided into non-castration-resistant prostate cancer (non-CRPC) or castration-resistant prostate cancer (CRPC). The criterion for CRPC was a testosterone level of below 50 ng/dl.

Consecutive prostate cancer patients who showed negative for skeletal metastasis on BS were enrolled between January and April 2013. These patients were followed up for at least 3 years and were confirmed negative for skeletal metastasis.

This retrospective study was approved by the local institutional review board.

### BS and BONENAVI analysis

**Fig. 1:**
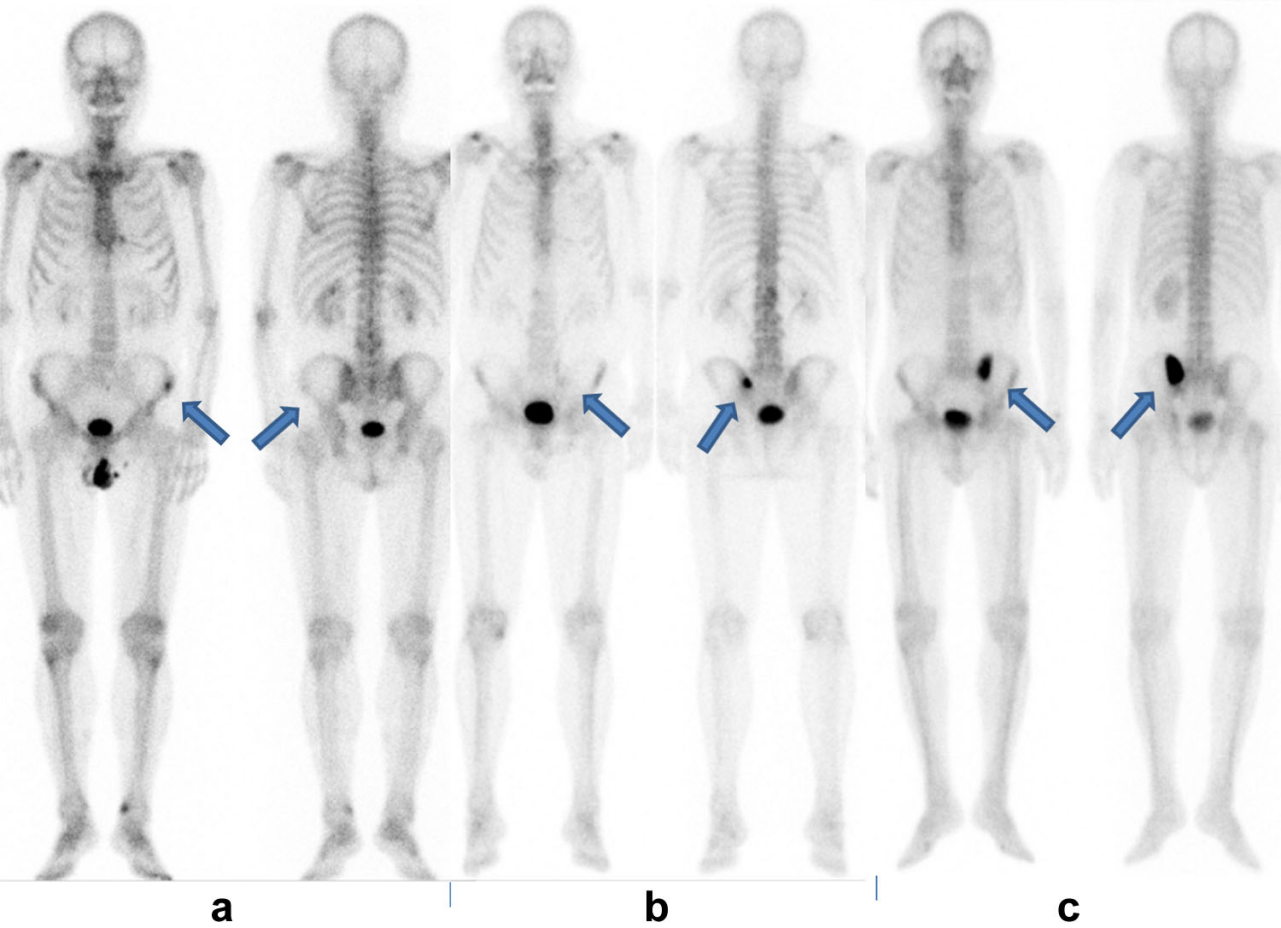
Bone scintigraphy uptake patterns are shown. Faint lesion uptake is shown on the right iliac wing (**a**), usual lesion uptake is shown on the left iliosacral site (**b**), and intense lesion uptake is shown on the iliosacral site (**c**). Faint uptake **a** is equal to, or slightly more intense than, that of the adjacent normal bones. Intense uptake **c** is seen in both anterior and posterior views. Usual uptake falls between faint and intense patterns. *Arrows* indicate hot spot sites

BS was performed about 3 h after the injection of ^99m^Tc-MDP. After obtaining anterior and posterior whole-body scans, local images including the pelvic axial view were acquired or, in some patients, bone SPECT/CT was performed as follow-up to the whole-body images. Patients in whom BS revealed metastasis were further analyzed. The bone metastatic burden of these patients, as shown on BS, was scored using Soloway’s extent of disease (EOD) system [9]. Briefly, EOD scores were decided on the basis of number and extent of metastases. The bone scans were divided into 5 EOD grades, *0* normal or abnormal due to benign bone disease, *I* number of bony metastases less than 6, each of which is less than 50% of vertebral body (one lesion about the size of a vertebral body would be counted as two lesions), *II* number of bone metastases between 6 and 20, size of lesions described above. *III* number of metastases more than 20 but less than a ‘super scan’, and *IV* ‘super scan’ or its equivalent, i.e., more than 75% of ribs, vertebrae, and pelvic bones. The intensity of skeletal metastatic uptake was visually graded as faint, usual, or intense on whole-body images. Figure 1 shows BS uptake patterns; faint uptake was visualized as a weak hot spot, whereas intense uptake was very strong uptake, which could be seen in both anterior and posterior views. Usual uptake was visualized somewhere between faint and intense uptake. This visual classification was independently performed by two radiologists-nuclear physicians, and 15 discordant cases were decided through discussion.

BONENAVI analysis was performed as previously reported [10]. Briefly, the CAD system “BONENAVI version 2.1.7” (FUJIFILM RI Pharma Co., Ltd., Tokyo, Japan) was used to analyze BS scans using anterior and posterior whole-body images. This BONENAVI system shows two imaging markers: ANN and a bone scan index (BSI). The ANN value indicates the probability of having skeletal metastasis. The range of ANN is 0–1, where “0” means no possibility of skeletal metastasis, and “1” means there is a high suspicion of having skeletal metastasis. The BSI indicates the tumor metastatic burden (proportion of bone metastatic area to whole body skeleton). In the present study, the ANN values were used.

### CT scans

CT scans were performed with a 2-mm thickness at 5-mm intervals.

**Fig. 2:**
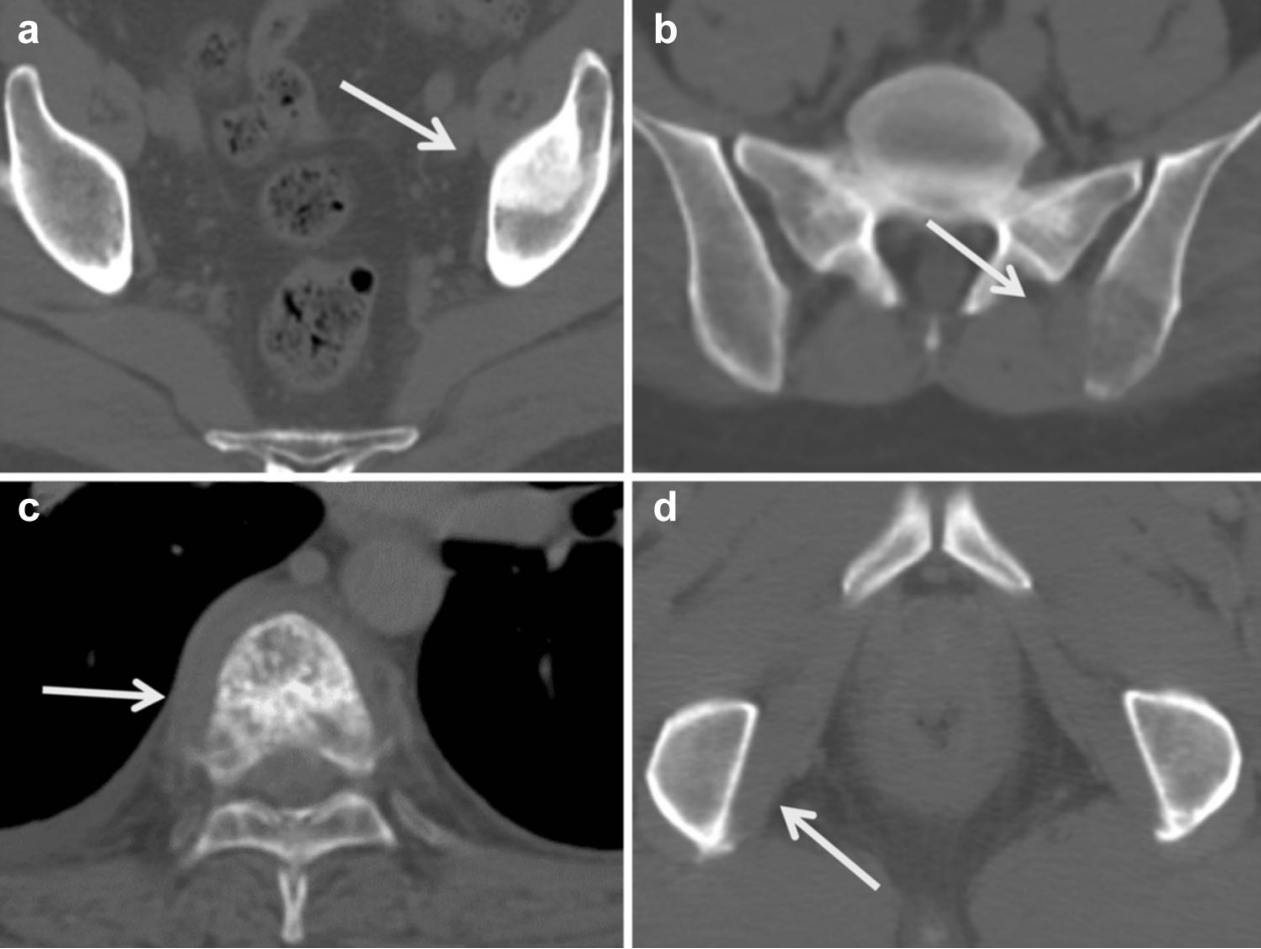
Representative examples of the various computed tomography (CT) morphologies: **a** osteoblastic pattern (left ilium); **b** osteolytic pattern (left ilium at the iliosacral site); **c** mixed osteolysis and osteoblastic pattern (lumbar vertebra); and **d** invisible pattern (right ischium). On a later CT scan of the invisible patient, the osteoblastic lesion has been developed. *Arrows* indicate osseous metastatic sites

The CT morphological classification of skeletal metastasis included the categories osteoblastic, osteolytic, mixed osteoblastic and osteolytic, and intertrabecular (invisible on CT). Figure 2 shows the morphologic lesion patterns on CT scans. This classification was independently performed by two radiologists-nuclear physicians, and discordant cases were decided through discussion. The confirmation of skeletal metastasis of intertrabecular or invisible on CT was performed by follow-up CT studies. All invisible lesions turned to osteoblastic or mixed CT appearance on later CT studies.

### Data analysis and statistical methods

The patient-base was evaluated as follows:

1. Receiver operating characteristics (ROC) curve analysis was performed using the patient’s ANN value in all patients.

Then, sensitivity and specificity were calculated with the cutoff level set at ANN = 0.5 for both skeletal metastasis positive and negative patients. The cutoff level was set at ANN = 0.5 throughout the study according to other Japanese studies [7, 8].

2. The sensitivity was also calculated as a function of the diagnostic setting (staging period, follow-up). Patients during the follow-up period were further divided into non-CRPC and CRPC groups. For the staging and follow-up (non-CRPC and CRPC) periods, we applied one-way analysis of variance (ANOVA) test followed by post hoc analysis using ANN values. The contingency table analysis was carried out using cutoff at ANN = 0.5.

Similar analyses were performed in regard to CT appearances, that is, osteoblastic, osteolytic, mixed, and intertrabecular (invisible) types, EOD scores (EOD = I, II, III, and IV), and BS uptake intensities (faint, usual, and intense).

3. False-negative and false-positive lesions were analyzed. Analysis of false-negative lesions was performed in patients with skeletal metastasis and ANN ≤ 0.5 value. False-negative patients were further divided into two groups of ANN 0–0.25, and 0.26–0.5 [7, 8]. Analysis of false-positive lesions was performed in patients with non-skeletal metastasis and ANN > 0.5. False-positive patients were also divided into two groups of ANN 0.51–0.75 and 0.76–1.00.

Statistical analysis software (SPSS version 24; IBM, Armonk, NY, USA) was used. A value of *p* < 0.05 was considered to indicate statistically significance. For ANOVA, Levene’s test for the homogeneity of variance was performed first. When the homogeneity of variance was assumed, a *t* test was followed by Turkey’s test. When the homogeneity was not assumed, Welch’s test was followed by the Games-Howell test. For contingency table analysis, if the more than 20% of cell boxes was expected numbered <5, Fisher’s exact test was used. Pearson’s Chi-square test was applied to the others.

## Results

This study included 226 patients with prostate cancer: 124 with skeletal metastasis and 102 without.

### ROC analysis with sensitivity and specificity

**Fig. 3:**
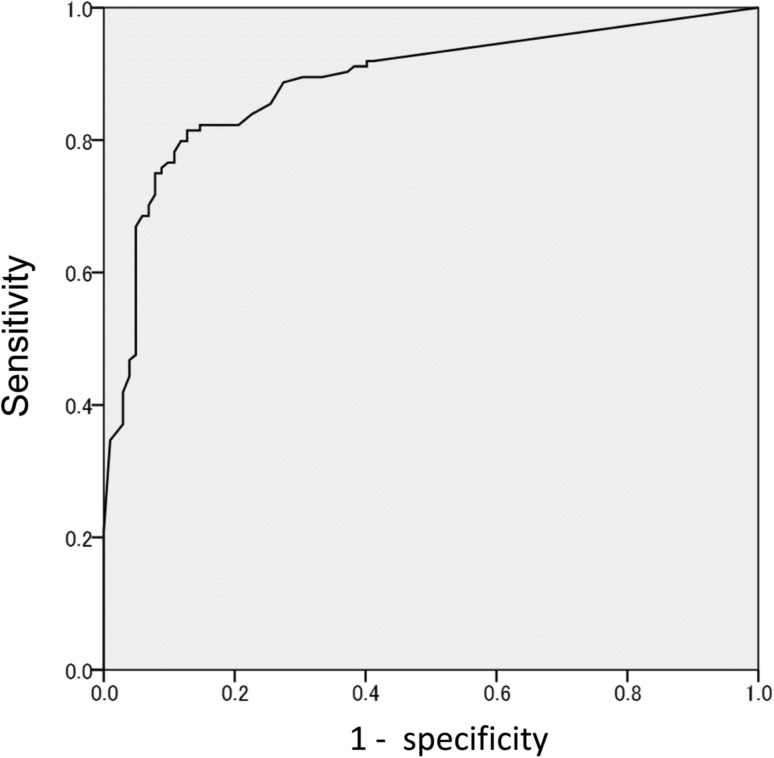
Receiver operating characteristics curve

The ROC curve is shown in Fig. 3. The area under the curve was 0.888 (95% confidence interval 0.843–0.932). The mean ANN values were 0.78 (SD = 0.29) for the skeletal metastasis patients and 0.22 (SD = 0.30) for the non-skeletal metastasis patients. Sensitivity was 82% (102/124), and specificity was 83% (84/101).

### Subgroups analysis

Tables 1, 2, 3 and 4 summarized the ANN values with ANOVA analyses and the contingency tables analyses using ANN cutoff value = 0.5 regarding diagnostic situation, CT appearance, EOD and BS uptake grade.

**Table 1:** ANN in skeletal metastasis among diagnostic situation

	*n*	ANN value*^,#^	Cross table (cutoff ANN = 0.5)^$^		
			ANN positive	ANN negative	Sensitivity (%)
Staging	57	0.89 ± 0.19	53	4	93
Follow-up					
Non-CRPC	42	0.65 ± 0.37	30	12	71
CRPC	25	0.76 ± 0.27	19	6	76

* Mean ± SD

^#^Welch test showed statistical significant difference (*p* = 0.001). Games–Howell test showed statistical significant difference between Staging and non-CRPC. Other pairs did not show statistical difference

^$^Fisher’s exact test showed *p* < 0.001 among groups

**Table 2:** ANN among CT appearance of skeletal metastasis

CT type	*n*	ANN value*^,#^	Cross table (cutoff ANN = 0.5)^$^		
			ANN positive	ANN negative	Sensitivity (%)
Blastic	83	0.76 ± 0.32	67	16	81
Lytic	7	0.79 ± 0.25	5	2	71
Mixed	19	0.90 ± 0.16	18	1	95
Invisible	15	0.77 ± 0.29	12	3	80

* Mean ± SD

^#^Welch test did not show statistical significant difference

^$^Fisher’s exact test did not show statistical significant difference

**Table 3:** ANN among EOD score of skeletal metastasis

EOD**	*n*	ANN value*^,#^	Cross table (cutoff ANN = 0.5)^$^		
			ANN positive	ANN negative	Sensitivity (%)
I	81	0.71 ± 0.32	60	21	74
II	23	0.94 ± 0.12	23	0	100
III	18	0.91 ± 0.18	17	1	94
IV	2	0.99 ± 0.00	2	0	100

* Mean ± SD

** *EOD* extent of disease

^#^Statistical significant difference between I and II, I and III, and I and IV (Welch’ test; *p* < 0.001 followed by Games–Howell test)

^$^Statistical significant difference was shown (Fisher’s exact test; *p* = 0.006)

**Table 4:** ANN among BS uptake grade of skeletal metastasis

BS uptake grade	*n*	ANN value*^,#^	Cross table (cutoff ANN = 0.5)^$^		
			ANN positive	ANN negative	Sensitivity (%)
Faint	32	0.57 ± 0.34	18	14	56
Usual	39	0.79 ± 0.31	34	5	87
Intense	53	0.90 ± 0.15	50	3	94

* Mean ± SD

^#^Statistical significant difference between faint and usual, and faint and intense (Welch’ test; *p* < 0.001 followed by Games–Howell test)

^$^Statistical significant difference was shown (Fisher’s exact test; *p* < 0.001)

As shown in Table 1, skeletal metastasis at follow-up period showed lower ANN values and lower sensitivity than those at staging with statistically significance.

Mixed type of CT appearance osseous metastasis showed high mean ANN value (mean ± SD, 0.90 ± 0.16) and high sensitivity (95%). Other types of CT appearance showed similar results (both ANN values and sensitivity; Table 2).

There was no statistical difference among the types of CT appearance.

Analysis of EOD differences showed that EOD = I patients had lower mean ANN value and lower sensitivity compared with EOD = II, III, IV patients. The ANN values and the sensitivity between EOD = I and EOD II-IV differed statistical significantly (Table 3).

The ANN value of for those with faint BS uptake was significantly lower than those for patients with usual or intense BS uptake. The sensitivity was also significantly lower in the patients with faint BS uptake group compared with that in those with usual or intense uptake (Table 4).

### Analysis of false-negative cases

Table 5 shows the 22 patients with false-negative results (ANN ≤ 0.5). Sub-groups of false-negative results were no suspicion (ANN 0–0.25) and less suspicion (ANN 0.26–0.5) according to Horikoshi [7]. Ten skeletal metastasis patients with no suspicion group (ANN 0–0.25) showed ANN = 0. All patients had a small bone tumor burden (EOD = I); most (9/10) had a solitary skeletal metastasis; most (9/10) were diagnosed during the follow-up period; and the BS uptake was generally faint (8/10). The 8 of 10 patients had lesions in pelvic bone: 2 in the ilium, 3 in the ischium, 1 in the sacrum, and 2 in the pubis. In 12 patients with less suspicion of skeletal metastasis (ANN 0.26–0.50), most patients (11/12) had small tumor burden (EOD = I). The BS uptake was higher (faint = 8, usual = 2, and intense = 2) than those of no suspicion group. Seven patients (7/12) had lesions in pelvic bone.

**Table 5:** False negative patients list

	Age	ANN	EOD	Lesion number	Situation	BS uptake	Site of osseous metastasis	Relation to urinary tract and comments
ANN 0–0.25								
1	67	0	I	1	Follow	Faint	L3	
2	77	0	I	1	Follow	Usual	L5	Mimics degenerative change
3	77	0	I	1	Follow	Faint	Ilium	
4	66	0	I	2	Follow CRPC	Faint	Ilium	
5	72	0	I	1	Follow	Faint	Ischium	
6	78	0	I	1	Follow	Faint	Ischium	
7	69	0	I	1	Staging	Faint	Ischium	
8	65	0	I	1	Follow	Faint	Sacrum	Right sacrum hot spot lies on ureter
9	55	0	I	1	Follow	Usual	Pubis	Left pubis hot spot lies on bladder
10	88	0	I	1	Follow	Faint	Pubis	Right pubis hot spot lies on bladder
ANN 0.26–0.50								
11	77	0.32	1	1	Follow CRPC	Faint	Th2	Mimics degenerative change
12	72	0.37	3	27	Follow	Faint	Ths	Multiple but very faint uptake
13	74	0.39	1	2	Staging	Faint	Pubis	
14	76	0.42	1	1	Follow	Intense	L4	Mimics degenerative change
15	39	0.43	1	1	Staging	Faint	Ilium	
16	78	0.43	1	1	Follow	Faint	Sacrum	
17	84	0.44	1	2	Follow CRPC	Intense	Ilium	Mimics degenerative change
18	72	0.44	1	1	Staging	Faint	Ilium	
19	74	0.45	1	1	Follow CRPC	Faint	Ilium	
20	67	0.45	1	1	Follow CRPC	Faint	Rib	Rib small faint
21	70	0.47	1	3	Follow	Usual	Ilium, L, rib	ilium (SI joint) usual but small
22	83	0.47	1	2	Follow CRPC	Usual	Ls	Mimics degenerative change

*ANN* artificial neural network, *EOD* extent of disease, *BS* bone scintigraphy, *CRCP* castration resistant prostate cancer, *L* lumbar vertebra, *Th* thoracic vertebra, *SI* sacroiliac

### Analysis of false-positive cases

Table 6 shows the 17 patients with ANN > 0.5. These patients were divided into seven patients of highly suspicion group (ANN 0.76–1.0) and 10 of suspicion group (ANN 0.5–0.74). These patients did not have skeletal metastasis, although BONENAVI analysis had indicated there was a possibility of skeletal metastasis. Most of the patients showed false-positive hot (red) spots on the vertebral column (16/17). Even though the BS hot spots existed at those sites, none of these patients were judged to have skeletal metastasis based on an experienced nuclear physician diagnosing those areas of uptake as degenerative changes. At their follow-up evaluations at least 3 years later, none of the patients had developed skeletal metastasis.

**Table 6:** False positive patients list

	Age		ANN	BSI	HSN	Hot (red) spot sites	Comments
ANN 0.76–1.00							
1	65	Follow	0.99	0.672	8	Ths,Ls	Degenerative change
2	73	Staging	0.98	0.39	5	Ths, Ls	Degenerative change
3	64	Staging	0.98	0.41	6	Ths, Ls, C, rt ilium	Degenerative change
4	76	Follow	0.95	0.292	4	Ths,Ls	Degenerative change
5	50	Follow	0.93	0.197	2	L5, rt scapula	L5 Degenerative change
6	76	Follow	0.78	0.074	2	Th, rt ilium	Degenerative change
7	77	Follow	0.77	0.073	3	Th1,12	Degenerative change
ANN 0.51–0.75							
8	67	Follow	0.74	0.023	1	Th12	Degenerative change
9	66	Staging	0.67	0.123	8	sternum, Th8	Degenerative change
10	70	Staging	0.65	0.178	2	Th7, lt SI	Degenerative change
11	77	Follow	0.63	0.266	3	Ths, Ls	Degenerative change
12	71	Staging	0.6	0.165	4	C7, bil-SC joint	Degenerative change
13	57	Staging	0.59	0.069	1	rib	Traumatic
14	71	Staging	0.56	0.164	1	ilium	Hip surgery
15	65	Staging	0.56	0.05	1	clavicle (distal)	Degenerative change
16	58	Staging	0.52	0.057	1	Th1	Degenerative change
17	81	Follow	0.51	0.034	3	Ths (lower)	Degenerative change

*ANN* artificial neural network, *BSI* bone scan index, *HSN* hot spot number, *L* lumbar vertebra, *Th* thoracic vertebra, *C* cervical vertebra, *SI* sacroiliac, *SC* sternoclavicular

## Discussion

BS has a high sensitivity for diagnosing skeletal metastasis and is an effective technique for the whole-body skeletal examination. However, interpretation of BS is performed visually. To address this problem, a quantitative or automatic approach was developed in the form of software using artificial intelligence to compute an ANN. This software offers the possibility of detecting skeletal metastasis. In addition, a BSI, using the bone tumor burden/whole skeleton ratio, was developed and used to diagnose bone metastasis and its follow-up [4–7]. This CAD software, commercially available as EXINI bone (EXINI Diagnostics, Lund, Sweden), is capable of learning to detect metastatic lesions on whole-body scans. The technology is able to identify hotspots, adjust whole-body intensity and quantify hotspot intensity, and classify hot spot lesions.

The Japanese version of this software, BONENAVI (FUJIFILM RI Pharma, Tokyo, Japan), was modified using a cohort of Japanese patients [8, 9], and revised [11]. In the present study, we used ANN obtained using BONENAVI version 2.1.7. to analyze diagnostic accuracy. Another index, BSI, shows the proportion of bone tumor in regard to the whole skeleton. The BSI has been reported to evaluate the therapeutic effect of various therapies on bone metastasis [12–14].

Skeletal metastasis from prostate cancer is usually osteoblastic, unlike other cancers, which are mostly osteolytic [15]. This study showed that 67% (83/124) of the lesions were osteoblastic in nature at the initial diagnosis: osteolytic type 6% (7/124); mixed type 15% (19/124); and invisible type 12% (15/124). That is, 82% (102/124) of patients (blastic + mixed) have an osteoblastic component on CT at the initial diagnosis of skeletal metastasis. From another viewpoint, 21% (26/124) of skeletal metastases have a lytic component (lytic + mixed). The importance of the lytic component is obvious in that anti-resorption medicines, bisphosphonate and anti-RANKL monoclonal antibody, are used to treat skeletal metastases of prostate cancer [16, 17]. We formerly reported that both formation and resorption markers increased in prostate cancer patients with skeletal metastasis [18].

The radiopharmaceutical used for BS accumulates at the bone-forming portion of the tumor, not at the tumor itself, and BS is weak to detect lytic metastases. Therefore, we thought that there might be some difference in the BONENAVI sensitivity among CT types. However, this study showed that the sensitivity of the lytic type did not have different ANN values (no statistically significant difference), and the sensitivity was only slightly low 71% (5/7), without statistical significance (Table 2). These findings mean that the ANN value and sensitivity of BONENAVI are robust for the various CT types. We formerly reported that the ANN and sensitivity of BONENAVI were not different among the CT types in breast cancer or lung cancer patients with skeletal metastasis [9].

Patients with EOD = I patients and those with BS weak uptake showed low ANN values, and the rate of identifying false-negative lesions was significantly high (Tables 3, 4). These findings indicated that limited spread and weak uptake were associated with low ANN values and a high rate of false-negative results. That is, it is difficult to detect the early phase of skeletal metastasis using BONENAVI.

Patients showed lower ANN values and a higher false-negative rate during the follow-up period than staging period (Table 1). Because the patients who completed the initial therapy were followed up carefully, especially with PSA measurement, early stage of skeletal metastasis was supposed to be diagnosed by BS–hence the high false-negative rate at follow-up.

Moreover, analysis of false-negative patients with ANN = 0 revealed that patients who had a solitary bone lesion in the pelvis (or adjacent to it) often had ANN = 0. Putative reasons were not only being solitary and/or weak uptake but also an overlay of urinary (ureteral and bladder) activity even though the uptake is usual.

Therefore, we routinely add a pelvic axial view or pelvic SPECT/CT to avoid this overlay problem. Because BONENAVI analysis employs the anterior and posterior whole-body images, urinary activity overlay seems to be difficult to overcome using the present methods. Our study also tried to determine the reason for the false-positive results. Patients with degenerative changes of the vertebra (mostly multiple) showed false-positive results.

In conclusion, prostate cancer patients with and without skeletal metastasis were assessed by BONENAVI (version 2.1.7) for BS. The sensitivity and specificity were 82% (102/124) and 83% (84/101), respectively. The sensitivities were not different among CT types, although they were statistically significantly different among EOD scores, BS uptake, and diagnostic situations. Patients with EOD = I, faint BS uptake, and low ANN values also had low sensitivity during the follow-up period. Patients with a solitary pelvic osseous metastasis with faint BS uptake or adjacent to the urinary system may show false-negative BONENAVI results.

## References

[1] Siegel RL, Miller KD, Jemal A (2015). Cancer statistics, 2015. CA Cancer J Clin.

[2] Cancer statistics in Japan. Cancer Information Service. 2013. http://ganjoho.jp.

[3] Hess KR, Varadhachary GR, Taylor SH, Wei W, Raber MN, Lenzi R (2006). Metastatic patterns in adenocarcinoma. Cancer.

[4] Imbriaco M, Larson SM, Yeung HW, Mawlawi OR, Edi Y, Venkatraman ES (1998). A new parameter for measuring metastatic bone involvement by prostate cancer: the bone scan index. Clin Cancer Res.

[5] Sadik M, Jakobsson D, Olofsson F, Ohlsson M, Suurkula M, Edenbrandt L (2006). A new computer-based decision-support system for the interpretation of bone scans. Nucl Med Commun.

[6] Sadik M, Hamadeh I, Nordblom P, Suurkula M, Hoglund P, Ohlsson M (2008). Computer-assisted interpretation of planar whole-body bone scans. J Nucl Med.

[7] Horikoshi H, Kikuchi A, Onoguchi M, Sjostrand K, Edenbrandt L (2012). Computer-aided diagnosis system for bone scintigrams from Japanese patients: importance of training database. Ann Nucl Med.

[8] Nakajima K, Nakajima Y, Horikoshi H, Ueno M, Wakabayashi H, Shiga T (2013). Enhanced diagnostic accuracy for quantitative bone scan using an artificial neural network system: a Japanese multi-center database project. Eur J Nucl Med Mol Imaging Res.

[9] Koizumi M, Wagatsuma K, Miyaji N, Murata T, Miwa K, Takiguchi T (2015). Evaluation of computer-assisted diagnosis system, BONENAVI version 2, for bone scintigraphy in cancer patients in routine clinical setting. Ann Nucl Med.

[10] Soloway MS, Hardeman SW, Hickey D, Raymond J, Todd B, Soloway S (1988). Stratification of patients with metastatic prostate cancer based on extent of disease on initial bone scan. Cancer.

[11] Koizumi M, Miyaji N, Murata T, Motegi K, Miwa K, Koyama M (2015). Evaluation of a revised version of computer-assisted diagnosis system, BONENAVI version 2.1.7, for bone scintigraphy in cancer patients. Ann Nucl Med.

[12] Dennis ER, Jia X, Mezheritskiy IS, Stephenson RD, Schoder H, Fox JJ (2012). Bone scan index: a quantitative treatment response biomarker for castration-resistant metastatic prostate cancer. J Clin Oncol.

[13] Mitsui Y, Shiina H, Yamamoto Y, Haramoto M, Arichi N, Yasumoto H (2012). Prediction of survival benefit using an automated bone scan index in patients with castration-resistant prostate cancer. BJU int.

[14] Alva A, Nordquist L, Daignault S, George S, Ramos J, Albany C (2017). Clinical correlates of benefit from radium-223 therapy in metastatic castration resistant prostate cancer. Prostate.

[15] Resnick D, Kyriakos M, Greenway G (2005) Skeletal metastasis, bone and joint imaging, 3rd edn. In: Resnick D, Kransdorf MJ (eds) Chapter 72. Philadelphia: Elsevier Saunders; 2005. pp. 1245–64.

[16] Saad F, Gleason DM, Murray R, Tchekmedyian S, Venner P, Lacombe L (2002). A randomized, placebo-controlled trial of zoledronic acid in patients with hormone-refractory metastatic prostate carcinoma. J Nat Cancer Inst.

[17] Fizazi K, Carducci M, Smith M, Damiao R, Brown J, Karsh L (2011). Denosumab versus zoledronic acid for treatment of bone metastases in men with castration-resistant prostate cancer: a randomized, double-blind study. Lancet.

[18] Maeda H, Koizumi M, Yoshimura K, Yamauchi T, Kawai T, Ogata E (1997). Correlation between bone metabolic markers and bone scan in prostatic cancer. J Urol.

